# Kaposi Sarcoma Masquerading as Methicillin-Resistant Staphylococcus aureus Soft Tissue Infection of the Foot in an Immunocompromised Transgender Female

**DOI:** 10.7759/cureus.79707

**Published:** 2025-02-26

**Authors:** Moamen Elhaddad, Alexander Carrillo-Kashani, Pegah Panirian, Aviv E Oren, B. David Massaband

**Affiliations:** 1 Foot and Ankle Surgery, Cedars-Sinai Medical Center, Los Angeles, USA; 2 Pathology and Laboratory Medicine, Cedars-Sinai Medical Center, Los Angeles, USA

**Keywords:** hiv aids, kaposi sarcoma management, mrsa infection, soft tissue masses, transgender female

## Abstract

Kaposi sarcoma (KS) is an acquired immunodeficiency syndrome-defining malignancy caused by human herpesvirus-8 (HHV-8), predominantly affecting immunocompromised individuals, particularly those with untreated or advanced human immunodeficiency virus (HIV). Here, we present the case of a 28-year-old homeless transgender female with untreated HIV (CD4 count = 175 cells/μL, HIV RNA = 221,000 copies/mL), latent syphilis, and methamphetamine use disorder, who presented with a five-month history of progressive left foot pain, ulceration, and swelling. Initial examination revealed extensive bilateral lower extremity ulcerative lesions, with a necrotic, violaceous mass on the left hallux and a similar lesion on the right medial ankle, raising suspicion for KS. However, the presence of purulent drainage and surrounding erythema suggested a superimposed bacterial infection. Bedside incision and drainage of the left hallux lesion was performed, followed by formal surgical debridement and excision of infected soft tissue masses. Histopathologic examination confirmed KS, characterized by spindle cell proliferation, slit-like vascular channels, and HHV-8 positivity on immunohistochemical staining. Wound cultures grew methicillin-resistant *Staphylococcus aureus* (MRSA), indicating a concurrent bacterial infection. The patient was initiated on antiretroviral therapy with Biktarvy (bictegravir/emtricitabine/tenofovir alafenamide) and treated with linezolid and amoxicillin-clavulanate for MRSA. Wound care and offloading led to significant improvement, with reduced drainage and progressive healing. This case underscores the diagnostic and therapeutic challenges of KS masquerading as MRSA soft tissue infection in immunocompromised patients. The overlapping clinical features of KS and bacterial infections, particularly in the lower extremities, highlight the importance of early biopsy, histopathologic confirmation, and a multidisciplinary approach to care. Furthermore, this case emphasizes the impact of social determinants of health, such as homelessness and substance use, on disease progression and treatment outcomes. Addressing these barriers is essential for improving care in vulnerable populations with complex, multifactorial conditions.

## Introduction

Kaposi sarcoma (KS) is an acquired immunodeficiency syndrome (AIDS)-defining malignancy caused by human herpesvirus-8 (HHV-8) and remains a significant concern in untreated or advanced human immunodeficiency virus (HIV) patients, particularly in men who have sex with men and immunocompromised individuals [1,2]. KS is a vascular neoplasm strongly associated with HHV-8 infection, a gamma-herpesvirus that primarily infects endothelial and spindle cells. HHV-8 promotes uncontrolled angiogenesis and inflammatory cytokine release, leading to the development of KS lesions. While HHV-8 infection alone is not sufficient to cause KS, immunosuppression, such as that seen in HIV/AIDS, significantly increases the risk of disease progression. Despite the overall decline in KS incidence following the widespread availability of antiretroviral therapy (ART), its prevalence remains disproportionately high in individuals with low CD4 counts (<200 cells/mm³) and persistent HIV viremia [3]. KS primarily affects the skin, mucosa, and visceral organs, with the lower extremities being a common site of cutaneous involvement due to dependent edema, chronic inflammation, and compromised lymphatic drainage [4].

Clinically, KS often manifests as violaceous plaques, nodules, or ulcerative lesions, which can mimic cellulitis and a broad spectrum of other dermatologic conditions, posing a diagnostic challenge [5,6]. The distinction between KS and bacterial soft tissue infections is critical in HIV-positive patients, as bacteria such as methicillin-resistant *Staphylococcus aureus* (MRSA) and other opportunistic pathogens are highly prevalent due to immune dysfunction, skin barrier compromise, and frequent environmental exposure [7]. Ulcerated KS lesions, particularly on the feet and lower extremities, are prone to secondary bacterial infections, further confounding clinical assessment and delaying oncologic diagnosis [8].

Delayed recognition of KS in the setting of a superimposed MRSA infection can lead to inappropriate antibiotic use, unnecessary surgical interventions, and deferral of ART initiation, ultimately worsening patient outcomes [9]. This case highlights the complex diagnostic and therapeutic challenges posed by coexisting KS and MRSA infection in a patient with advanced HIV, emphasizing the need for a multidisciplinary approach involving infectious diseases, oncology, and podiatric surgery for optimal management.

## Case presentation

A 28-year-old transgender male to female with a history of untreated HIV (CD4 count = 175 cells/μL, HIV RNA = 221,000 copies/mL), latent syphilis, and methamphetamine use disorder presented to the emergency department with a five-month history of progressive left foot pain, ulceration, and swelling. The patient reported worsening discomfort, difficulty ambulating, and purulent extremely malodorous drainage. She was currently homeless and had been lost to HIV care despite a prior brief initiation of ART, which she discontinued due to a lack of follow-up.

**Figure 1:**
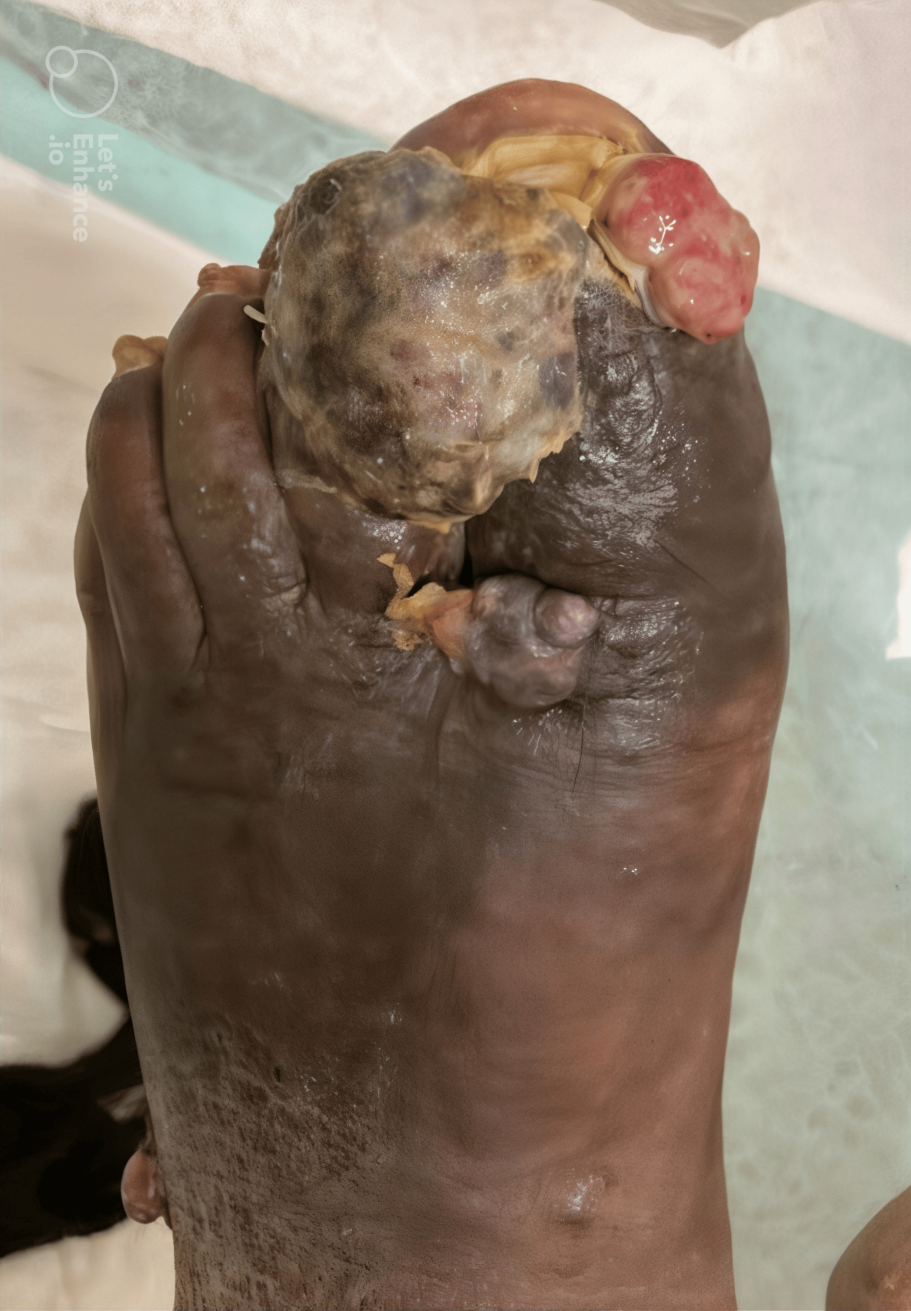
Left hallux. Extensive ulcerative and exophytic lesions involving the left hallux, with the most prominent lesion on the distal lateral hallux, presenting as a friable, necrotic mass with violaceous discoloration. A smaller, infected lesion is also visible on the distal medial hallux.

**Figure 2:**
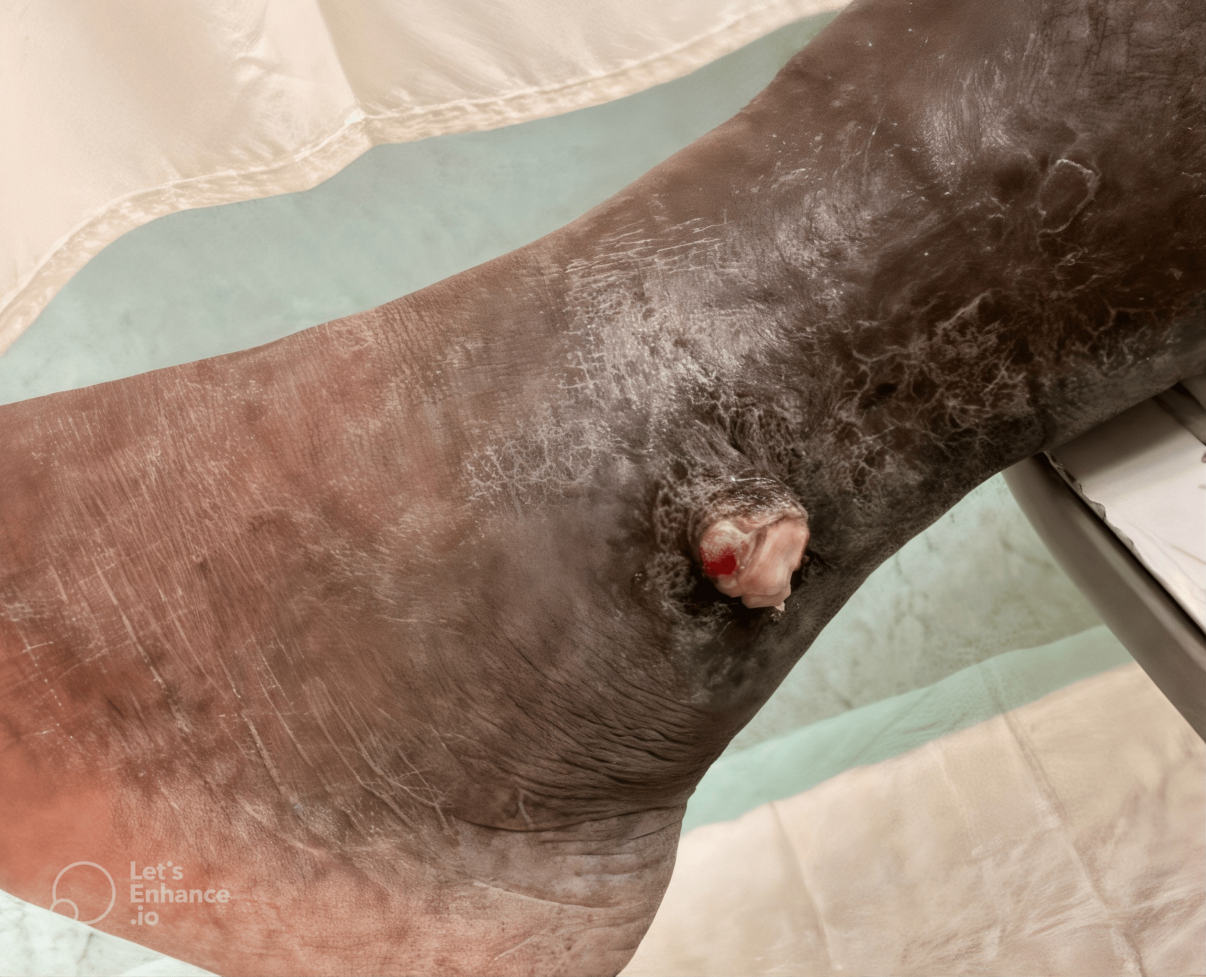
Right medial ankle. Nodular ulcerated lesion on the right medial ankle, extending across the medial malleolus, exhibiting violaceous discoloration and surrounding erythema.

**Figure 3:**
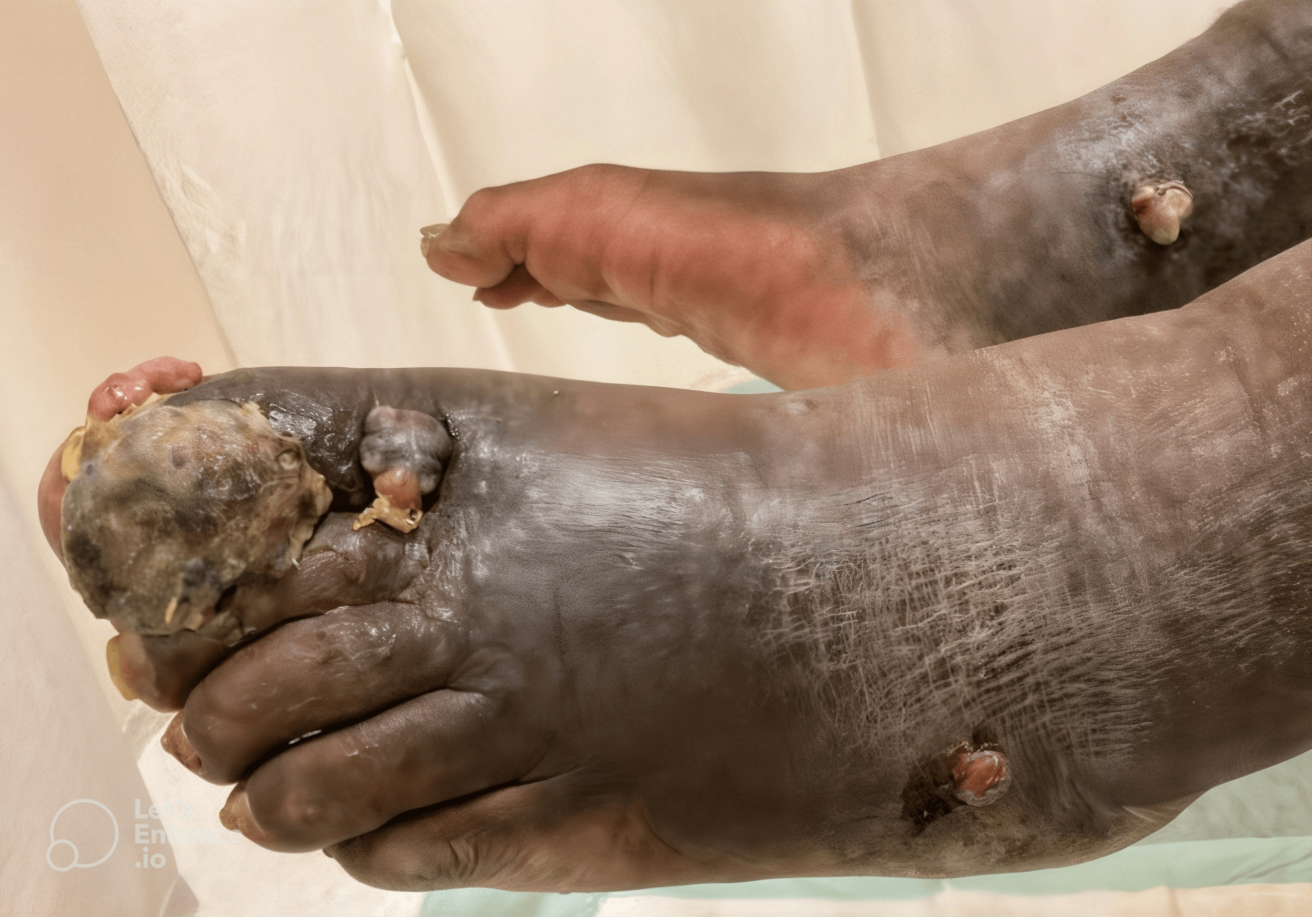
Left dorso-lateral foot. Additional non-infected exophytic lesions located on the proximal lateral left hallux and the left lateral-dorsal midfoot.

On initial examination, the patient was afebrile but exhibited extensive bilateral lower extremity ulcerative lesions, with the most pronounced findings localized to the left hallux and the right ankle. The left distal lateral hallux lesion was the largest, presenting as an exophytic, friable, necrotic mass with violaceous discoloration, surrounded by areas of induration and maceration (Figure 1). A smaller but infected lesion was also noted on the left distal medial hallux (Figure 1). Additionally, a nodular ulcerated lesion was identified on the right medial ankle, extending across the medial malleolus, exhibiting a similar violaceous hue with surrounding erythema (Figure 2). Other non-infected exophytic lesions were observed on the proximal lateral left hallux and the left lateral-dorsal midfoot (Figure 3). Laboratory findings included a white blood cell count of 8.69 × 10⁹/L, erythrocyte sedimentation rate of 130 mm/hour, and C-reactive protein (CRP) of 86.6 mg/L, indicating a significant inflammatory response. Given the chronicity of symptoms and the patient’s immunocompromised status due to HIV, an underlying neoplastic process such as KS was strongly considered, although superimposed bacterial infection remained a significant concern, particularly in the left hallux and right medial ankle lesions.

Given the severity of the left distal lateral hallux lesion, bedside incision and drainage (I&D) was performed in the emergency department, yielding purulent material. Partial excision of the exophytic lesion was undertaken, and the specimen was submitted for histopathologic evaluation. Empiric broad-spectrum antimicrobial therapy with linezolid and amoxicillin-clavulanate was initiated to provide coverage against gram-positive and anaerobic pathogens, pending culture results. The patient was subsequently admitted for further evaluation and management. Due to concerns regarding progressive infection involving the left hallux and right ankle, contrast-enhanced computed tomography (CT) imaging of both lower extremities was obtained.

**Figure 4:**
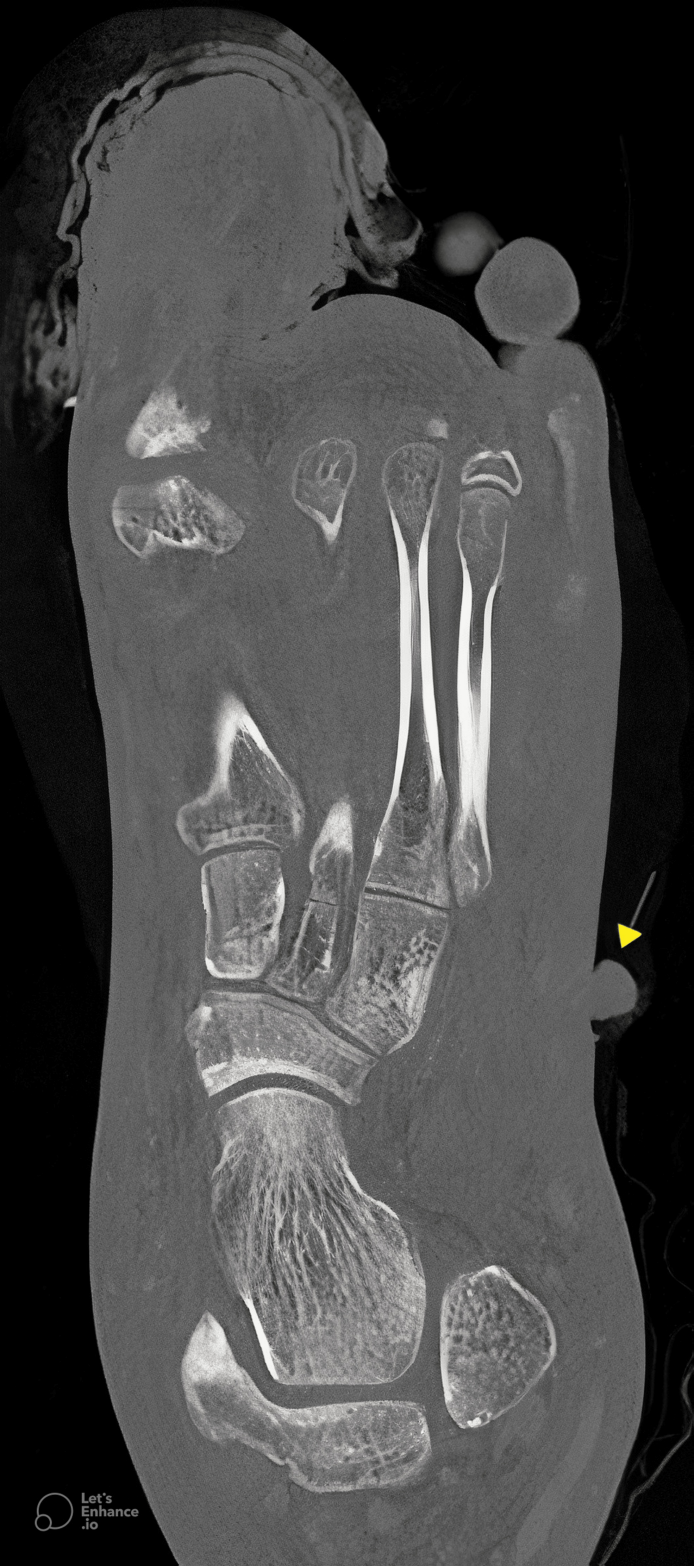
Axial CT of the left foot. Axial CT of the left foot demonstrating a small soft tissue mass extending from the lateral aspect of the foot (arrow). Extensive subcutaneous edema and thickening of the soft tissue planes are observed throughout the foot, especially the hallux area.

**Figure 5:**
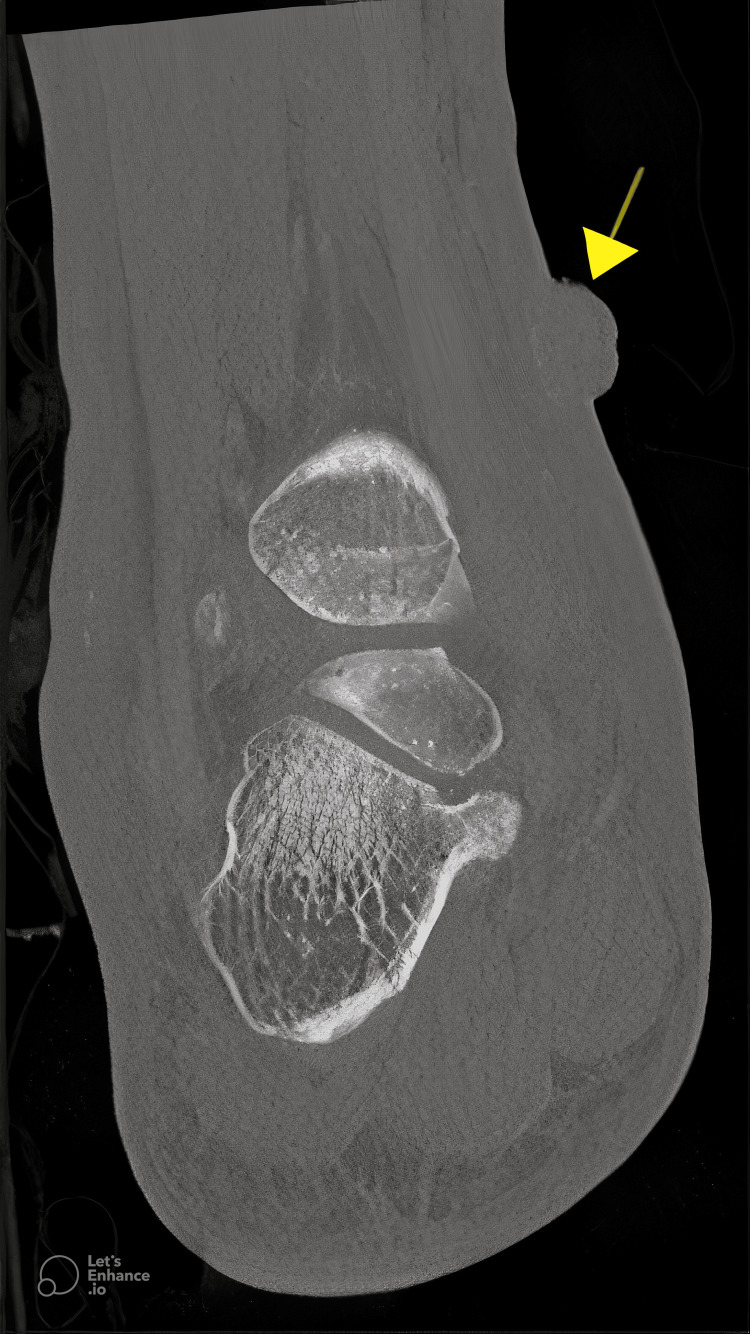
Coronal CT of the right ankle. Coronal CT of the right ankle demonstrating a small soft tissue mass extending from the medial aspect of the ankle (arrow). Extensive soft tissue swelling and subcutaneous edema are evident throughout the right lower extremity, with diffuse thickening of the subcutaneous fat planes, consistent with inflammatory changes.

CT imaging of the left foot with contrast revealed a left great toe wound with moderate soft tissue swelling and subcutaneous edema consistent with cellulitis, extending into the dorsum of the foot, ankle, and distal lower leg, with no evidence of an underlying abscess or osteomyelitis (Figure 4). Additionally, a 1.7 cm hyperdense subcutaneous mass was identified in the left lateral midfoot near the base of the fifth metatarsal, along with a 1.0 cm pedunculated skin lesion on the dorsal-lateral midfoot (Figure 4). CT imaging of the right ankle with contrast demonstrated a soft tissue mass extending from the medial ankle, measuring 2.3 × 1.9 × 1.9 cm (Figure 5).

**Figure 6:**
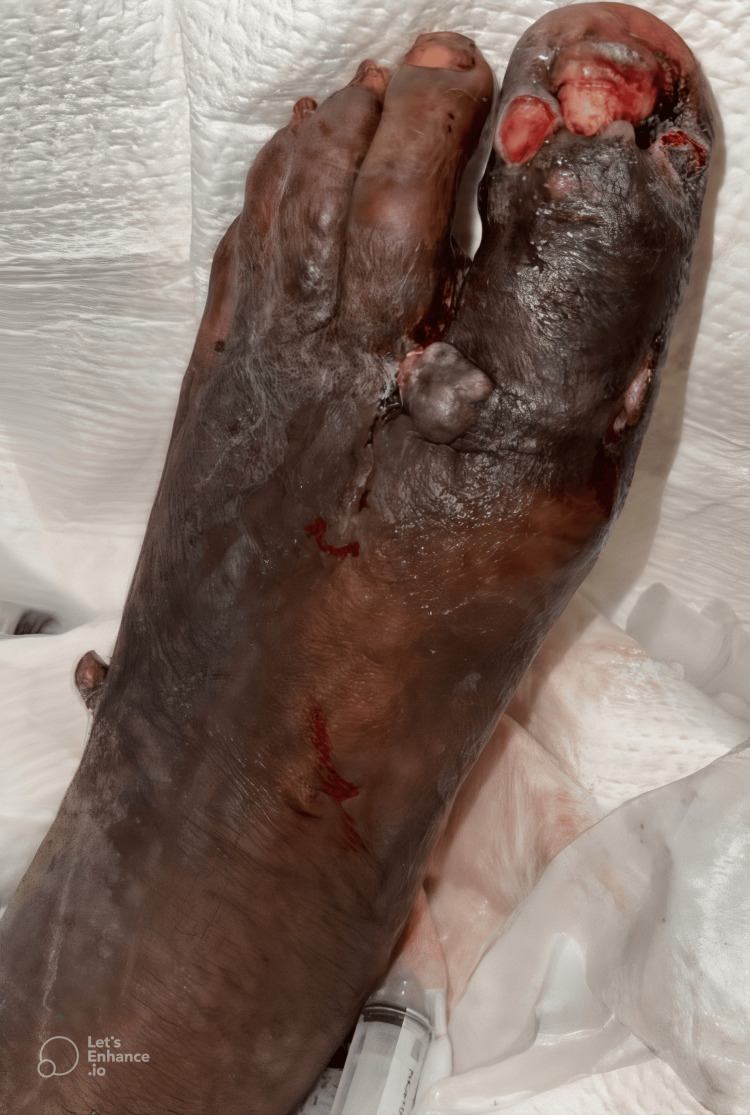
Left hallux: postoperative day one. Left hallux on postoperative day one showing the surgical sites following excision of the lesions, with surrounding soft tissue edema and hemorrhagic changes.

**Figure 7:**
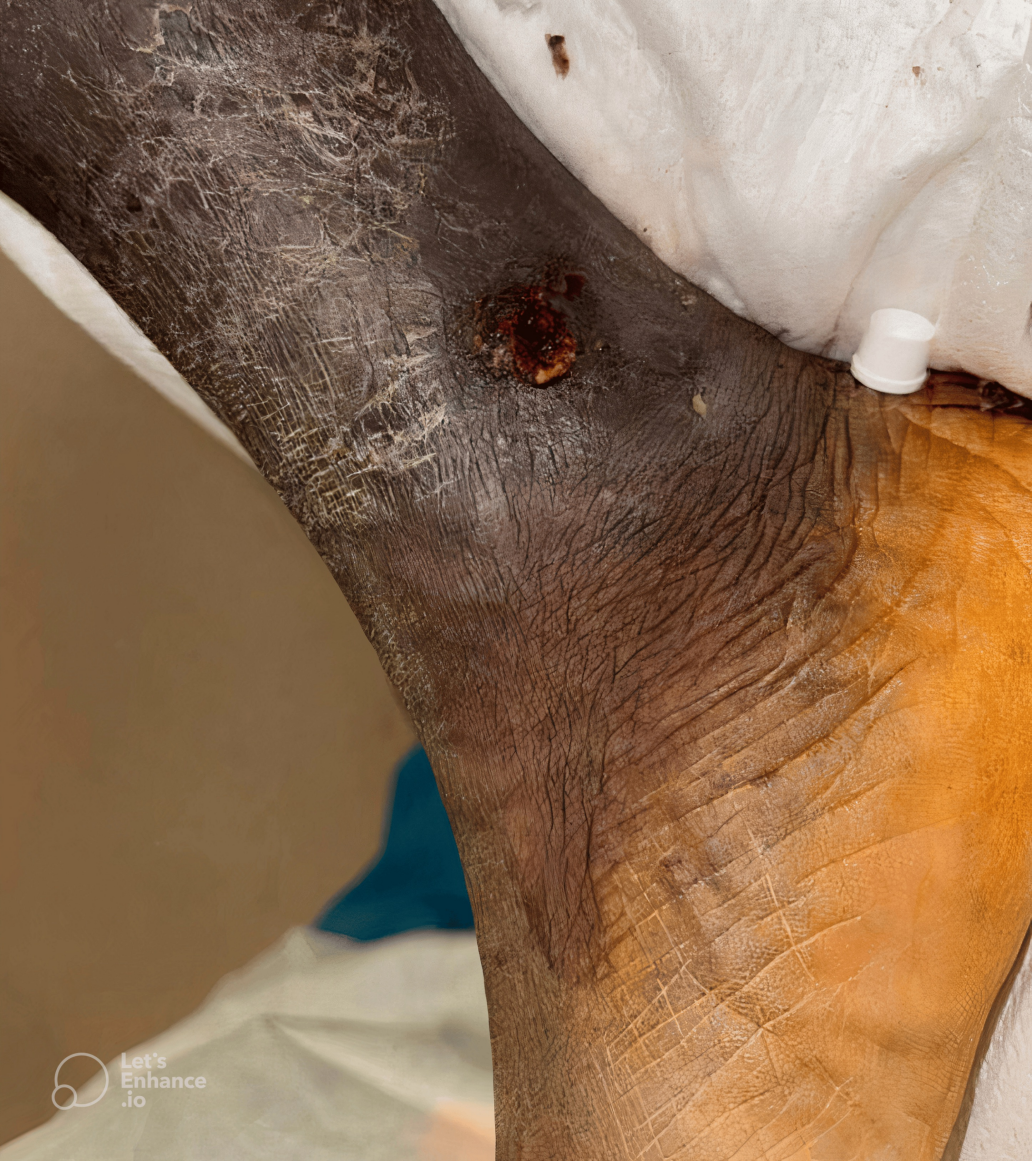
Right medial ankle: postoperative day one. Right medial ankle on postoperative day one showing the surgical site following excision of the lesions, with hemorrhagic changes.

On the following day, the surgical team performed a formal bilateral lower extremity I&D with excision of infected soft tissue masses, including the left distal medial hallux lesion, the remaining distal lateral hallux lesion, and the right medial ankle lesion, in the operating room. Other non-infected exophytic lesions at the proximal lateral left hallux and left lateral-dorsal midfoot were left intact (Figure 6). Intraoperatively, both left hallux lesions were extensively necrotic, with involvement extending into the surrounding soft tissue and dorsum of the foot. The right medial ankle lesion was similarly ulcerated and firmly adherent to the subcutaneous tissue. All infected lesions bilaterally were excised and submitted for biopsy. No overt signs of osteomyelitis were identified intraoperatively. The surgical team proceeded with aggressive debridement of necrotic tissue bilaterally, intraoperative culture collection, copious irrigation, application of antibiotic powder, and wound coverage with sterile dressings (Figures 6, 7).

**Figure 8:**
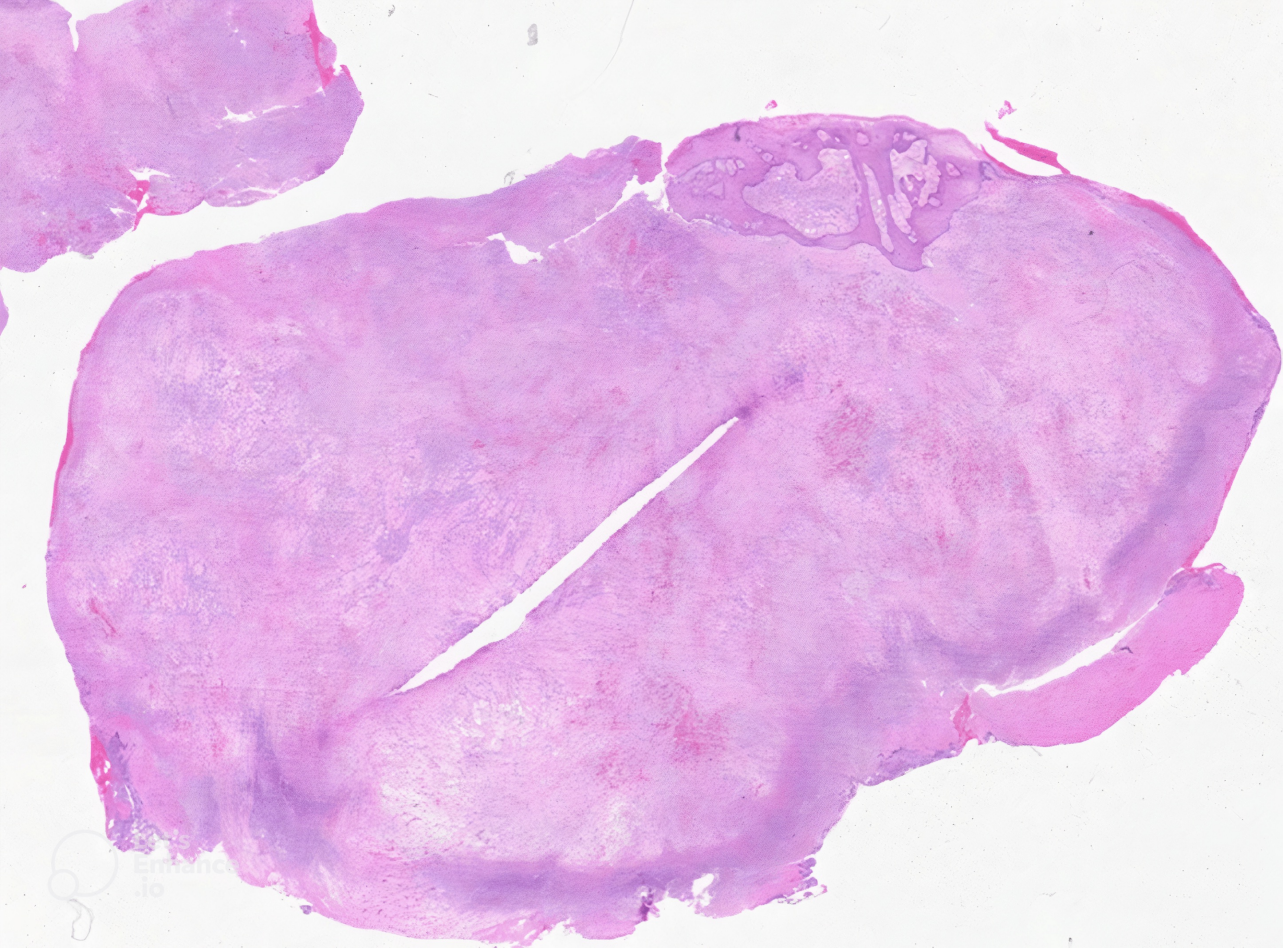
Low-power histopathologic view. Low-power histopathologic view of a dermal-based mass with overlying ulceration and fibrin deposition.

**Figure 9:**
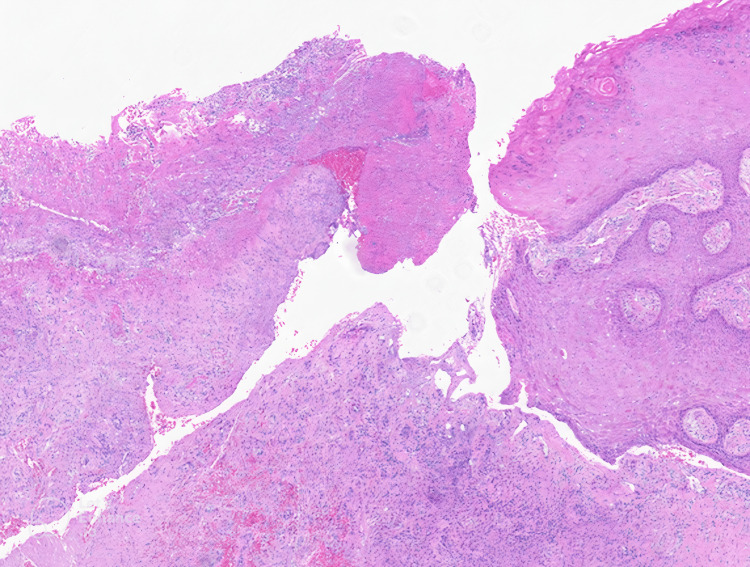
Medium-power histopathologic view. Medium-power histopathologic view reveals that the mass is composed of intersecting fascicles of uniform spindle cells.

**Figure 10:**
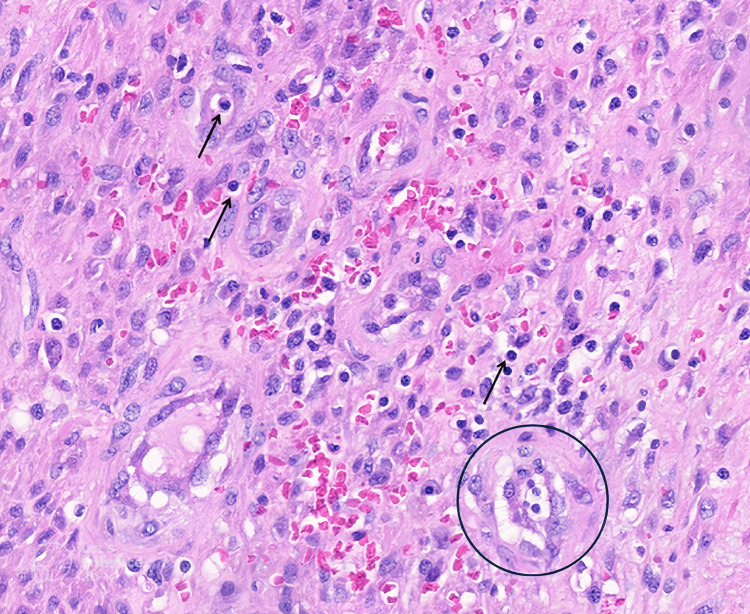
High-power histopathologic view 1. High-power histopathologic view demonstrates a spindle cell tumor with jagged, interconnected slit-like vascular channels, characteristic of Kaposi sarcoma (KS). Extravasated erythrocytes and scattered plasma cells (arrow) are observed within the tumor microenvironment. Notably, the promontory sign, a diagnostic hallmark of KS, is evident, characterized by a blood vessel forming within another blood vessel (circle).

**Figure 11:**
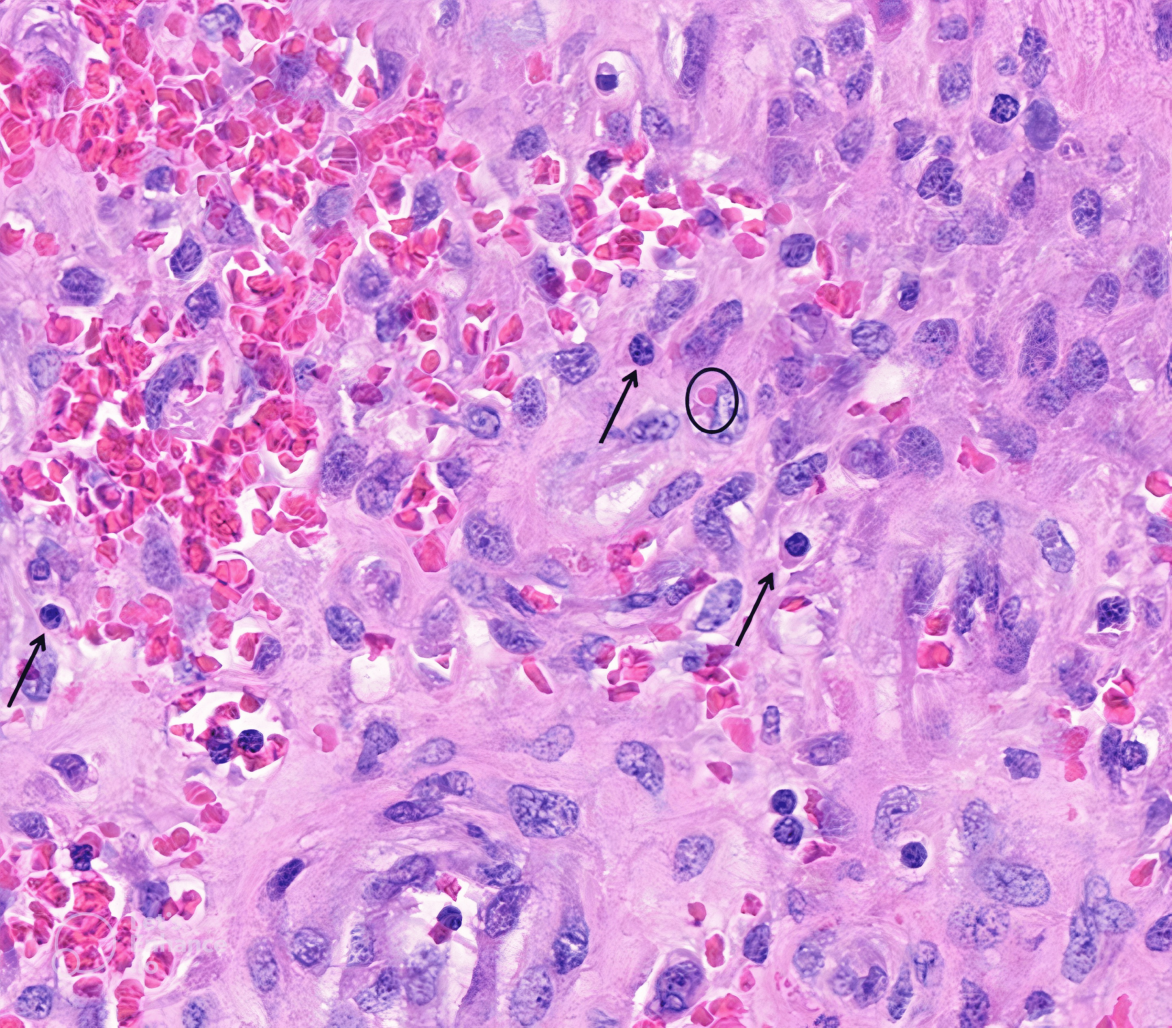
High-power histopathologic view 2. High-power histopathologic view reveals a spindle cell neoplasm with jagged, interconnected slit-like vasculature, characteristic of Kaposi sarcoma. Extravasated erythrocytes and scattered plasma cells (arrows) are present within the tumor microenvironment. Additionally, intracytoplasmic hyaline globules (circle) can be identified.

**Figure 12:**
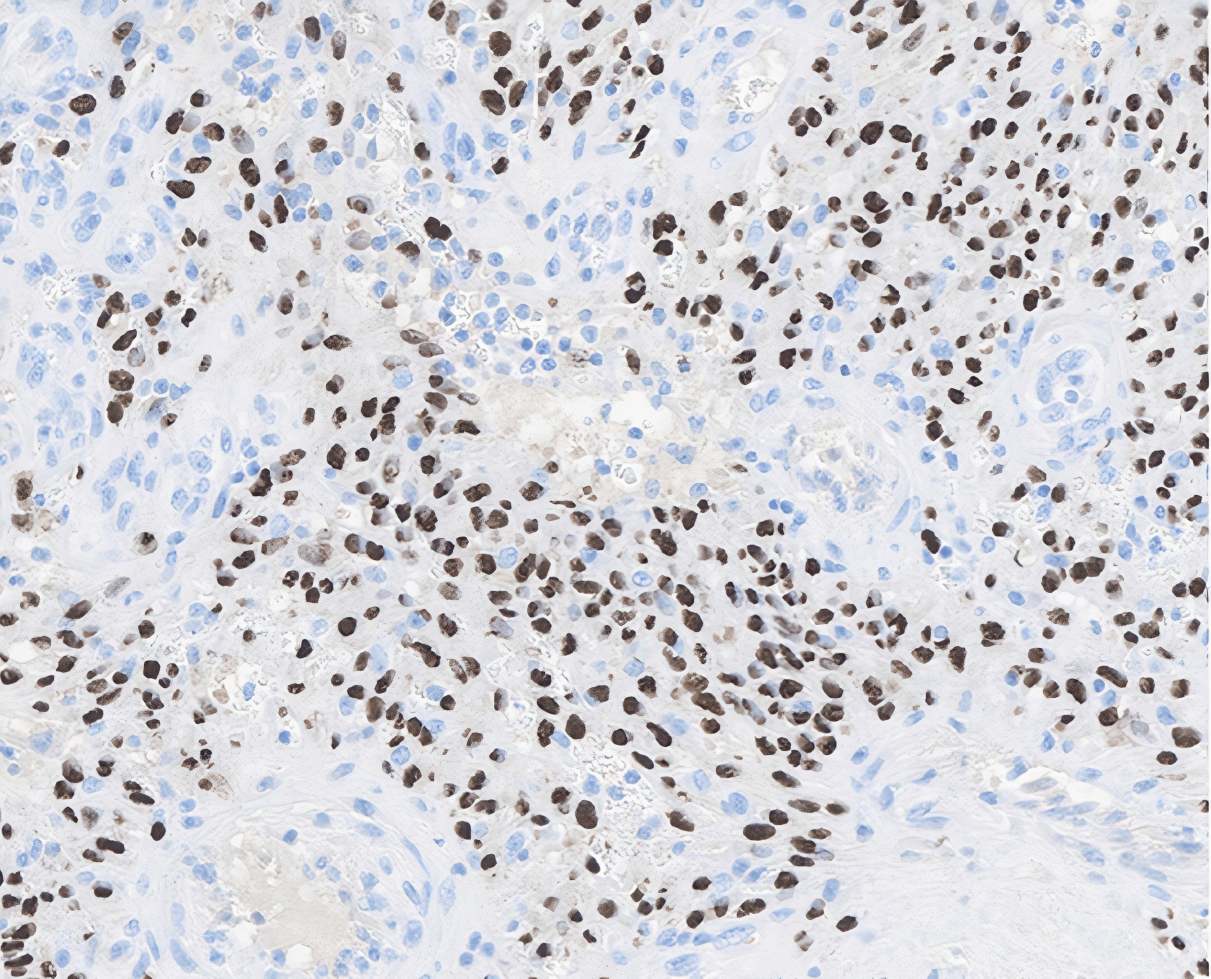
Human herpesvirus-8 (HHV-8) (13B10) immunohistochemical stain. HHV-8 (13B10) immunohistochemical stain demonstrates latent nuclear antigen (LANA) protein expression in endothelial and spindle cells, confirming Kaposi sarcoma.

Histopathologic examination of the excised lesions revealed spindle cell proliferation, slit-like vascular channels, and extravasated red blood cells, consistent with KS, with HHV-8 positivity on immunohistochemical staining, confirming the diagnosis (Figures 8-12). Additionally, wound cultures later grew MRSA, supporting the presence of a superimposed bacterial infection.

Following the confirmation of KS, the infectious diseases and oncology teams were consulted. Given her advanced HIV status, the patient was initiated on bictegravir-emtricitabine-tenofovir alafenamide for ART re-initiation. Oncology recommended ART as the primary treatment for KS, with close outpatient follow-up for lesion progression. Trimethoprim-sulfamethoxazole was continued for *Pneumocystis jirovecii* pneumonia prophylaxis, and penicillin G benzathine was scheduled for completion of her latent syphilis treatment.

Wound care and offloading resulted in significant improvement, with decreased erythema and reduced drainage observed within five days. By day 14, granulation tissue had formed at the surgical sites, indicating progressive healing. The patient completed a course of linezolid and continued amoxicillin-clavulanate for MRSA, with clear signs of infection resolution. Inflammatory markers, such as CRP, demonstrated a downward trend by day seven of antibiotic therapy. Following clinical stabilization, she was discharged with referrals to podiatry, oncology, and HIV primary care for ongoing wound monitoring, oncologic follow-up, and ART optimization.

## Discussion

This case highlights the diagnostic and therapeutic challenges of KS masquerading as an MRSA soft tissue infection in an immunocompromised patient with advanced HIV. The coexistence of oncologic and infectious pathology in the foot underscores the complexity of managing dual diagnoses in HIV-positive individuals, particularly when social determinants of health exacerbate disease progression. The clinical overlap between KS and bacterial infections often leads to diagnostic delays, emphasizing the need for early biopsy, histopathologic confirmation, and a multidisciplinary approach to care.

KS, an HHV-8-driven angioproliferative neoplasm, is a well-recognized AIDS-defining malignancy. While classic KS lesions are violaceous, non-tender plaques or nodules, atypical presentations, such as ulcerative, necrotic, or exophytic lesions, can closely mimic bacterial soft tissue infections [1,10]. In this patient, the left hallux lesion exhibited necrotic, friable tissue with surrounding erythema and purulent drainage, initially suggesting MRSA infection (Figure 1). However, the violaceous hue and chronicity of the lesions raised suspicion for KS, prompting histopathologic evaluation. The overlapping clinical features of KS and MRSA infection are particularly problematic in immunocompromised patients, where superimposed bacterial colonization is common because of immune dysfunction and skin barrier compromise, particularly in ulcerated KS lesions [7]. The lower extremities, including the feet, are highly susceptible due to dependent edema, compromised lymphatic drainage, and frequent trauma, which facilitate both KS progression and bacterial superinfection [4].

Histopathology remains the gold standard for diagnosing KS, particularly in cases with overlapping infectious features. The characteristic findings of spindle cell proliferation, slit-like vascular channels, and extravasated red blood cells were pathognomonic in this case [11]. HHV-8 immunohistochemical staining provided definitive confirmation, distinguishing KS from other vascular neoplasms or reactive processes [12]. The importance of early histopathologic evaluation cannot be overstated in patients with ulcerated lesions, as it allows for timely differentiation between infection, malignancy, and other inflammatory processes. Early biopsy and immunohistochemical analysis are critical to avoid diagnostic delays, particularly in the setting of coexisting infections. In this patient, surgical debridement of the hallux lesion facilitated histopathologic analysis, ultimately leading to the correct diagnosis and oncologic intervention. 

MRSA colonization and infection are highly prevalent in HIV patients, occurring at significantly higher rates than in immunocompetent individuals [7]. Studies have demonstrated that people living with HIV are at an increased risk of recurrent and invasive MRSA infections, particularly when chronic wounds or ulcerated lesions are present [13]. In this case, MRSA was confirmed through culture, explaining the inflammatory response and purulent drainage, which initially obscured the underlying oncologic process.

The management of coexisting KS and MRSA infection in this patient required a coordinated, multidisciplinary strategy involving podiatric surgery, infectious diseases, oncology, and HIV care. ART serves as the cornerstone of KS management, with studies indicating that lesion regression occurs in up to 80% of patients within several months of initiation, coinciding with an increase in CD4 counts and a reduction in viral load [14-16]. In this case, the patient was started on bictegravir-emtricitabine-tenofovir alafenamide, a first-line once-daily integrase inhibitor-based regimen, which is preferred for its efficacy, tolerability, and high barrier to resistance in treatment-naïve patients [17]. The presence of MRSA necessitated targeted antibiotic therapy, with linezolid and amoxicillin-clavulanate providing broad coverage for skin and soft tissue infections. The resolution of infection following antibiotic therapy highlights the importance of addressing superimposed bacterial colonization in ulcerated KS lesions.

Podiatric surgery played a pivotal role in this case, as surgical debridement of the necrotic hallux lesion facilitated both infection control and histopathologic diagnosis. Surgical intervention is particularly important in lower extremity KS, where lesions are prone to ulceration, necrosis, and secondary infection. Following surgery, wound care and offloading strategies led to progressive improvement, with decreased erythema, reduced drainage, and formation of granulation tissue. While ART is the primary treatment for KS, persistent or extensive disease may require additional modalities, such as liposomal doxorubicin or radiation therapy [18,19]. In this case, oncology recommended close outpatient follow-up to monitor lesion progression and determine the need for further intervention.

This case also highlights the profound impact of social determinants of health on HIV-related outcomes. The patient’s homelessness, methamphetamine use, and lack of consistent medical care contributed to disease progression, ART discontinuation, and delayed diagnosis of KS. Studies have consistently shown that housing instability and substance use disorders are major predictors of ART non-adherence, leading to higher rates of opportunistic infections and malignancies [20].

This case reinforces the importance of integrated care models that address both medical and social barriers to treatment. Patient navigation programs, housing support initiatives, and substance use interventions are critical to reducing HIV-related disparities and improving long-term outcomes in marginalized populations [20]. In this patient, lost follow-up and ART discontinuation led to a CD4 count decline to 175 cells/μL and uncontrolled viremia, creating an optimal setting for KS development. Addressing these factors is essential in preventing future complications and ensuring continuity of care.

## Conclusions

This case highlights the diagnostic and therapeutic challenges of coexisting KS and MRSA infection in an immunocompromised patient with advanced HIV. The overlapping clinical features of KS and bacterial infections, particularly in the lower extremities, underscore the importance of maintaining a high index of suspicion for malignancy in this population. Early biopsy, histopathologic confirmation, and a multidisciplinary approach are critical to ensuring accurate diagnosis and optimal management. Furthermore, this case emphasizes the need for integrated healthcare models that address both medical and social determinants of health. By addressing barriers to care and providing comprehensive, patient-centered treatment, clinicians can improve outcomes for vulnerable populations with complex, multifactorial conditions.
